# Cultural adaptation and transitions within international higher education: University students’ experiences of studying abroad during the 2020 Coronavirus pandemic

**DOI:** 10.1371/journal.pone.0308134

**Published:** 2024-10-24

**Authors:** Teneisha Ellis, Corinne Jola, Andrea Cameron

**Affiliations:** Department: Psychology, School of Applied Sciences, Abertay University, Dundee, Scotland, United Kingdom; Far Eastern University - Manila, PHILIPPINES

## Abstract

This qualitative study explored students’ lived experiences of higher education study abroad programs during the Covid-19 pandemic. Studying individual experiences in extraordinary circumstances like a pandemic can reveal personal, organisational and process-related resilience relevant to understanding and planning future events. Online semi-structured interviews with thirteen international students from four continents *(North America*, *South America*, *Europe and Asia)* were conducted amidst the pandemic in the Spring of 2021. Transition Theory underpinned the study. Interviews with students were used to explore personal strategies adopted to support the transition to virtual platforms during the pandemic. Through Interpretative Phenomenological Analysis, four response categories were identified: Functioning in Ambiguity, The Importance of Language and Culture, Reshaping Community, and Alternative Experiences in Intercultural Connectivity. Participants’ responses delved into aspects of acculturation, culture shock and resiliency amidst the disruption. These findings may inform future practices for international professionals supporting students studying abroad.

## Introduction

International student mobility (ISM) programs offer valuable opportunities for personal growth, cultural immersion and expanding on emotional and intercultural competencies. The number of students who participated in international mobility programs and studied abroad outside their country of origin was at an all-time high before the Covid-19 pandemic. United Nations Educational, Scientific and Cultural Organization (UNESCO) [1] reported that over 5 million individuals participated in international student mobility programs worldwide at that point. The Institute of International Education in the United States (IIE) [2] outline that exchange strategies should enable and support students in experiences of cultural adaptation and immersion into new language learning environments and facilitate the intrapersonal growth of participants. Indeed, studying abroad can involve learners from multiple countries and cultures coming together within program groups; accordingly, they must integrate into an unfamiliar environment away from their home countries [3, 4], which in effect provides them with excellent opportunities for personal development and the potential to enhance their intercultural sensitivities [5, 6]. However, DeLoach et al. [7] emphasized that the depth of the cultural immersion and duration of international programs can be impacted by the slightest changes in one’s environment, and the Covid-19 Pandemic was a significant interruption. In 2020, Covid-19 restrictions were introduced that curtailed travel, including to university campuses, necessitating online teaching for most students, with some international students unable to return to their home country [8]. The move to online teaching and learning on virtual platforms happened with little time for training or preparation [9]. Moving to a different learning environment, specifically online, and discovering and functioning in new cultural norms posed additional challenges for international students. Students had to adapt independently to an unfamiliar cultural space while learning about new education systems [10]. Understanding the lived experiences of students who went through this transition is critical for universities that may have to reimagine academic structures in future socially disruptive situations. This study aimed to explore two key questions: 1) What are the lived experiences of university students who transitioned to virtual learning on international programs during the 2020 Covid-19 Pandemic and 2) What can be learned from these experiences to improve future international exchange programming? The aim of this study was not to evaluate Covid-related online education systems but instead, it was to contribute to the dialogue concerning the perception of university students’ development during study abroad programs, specifically in times of social disruptions. It is also mean to understand the participants’ lived experiences as they navigated their transition to an alternate learning environment online. This study thus aimed to examine how students processed the pandemic-driven changes that occurred to their anticipated study abroad experiences. This research uses the framework of Transition Theory to explore international students’ perspectives as they transitioned to online learning.

## Literature review

The global pandemic significantly disrupted international student mobility (ISM) programming [11]. As such, it also greatly impacted the internationalization of higher education as many universities shifted to online platforms [12]. According to OECD [13], in 2020, when the pandemic began, 20% of the UK’s student body consisted of international students, both undergraduate and postgraduate, among whom the first author was a PhD student. Study Abroad programming options has grown significantly since the 1960’s [14], transcending the notion that only a select few can choose to participate [15]. The influential European Region Action Scheme for the Mobility of University Students (ERASMUS) program was established in 1987 to promote cooperation between higher education institutions across Europe, and as of the date of their commissioned report, sent over 9 million students on their programs [16]. In the 21st century, the landscape of study abroad has transformed, as evidenced by the numerous programming options now available, catering to varying program durations or degree opportunities [7, 17]. These options include traditional and nontraditional study destinations and other credit-bearing opportunities, including internships, intended to accommodate the interests of increasingly diverse, globally minded student cohorts [18–20]. In addition, Kim and Lawrence [21] note that studying abroad helps students grow more confident and mature, gaining a deeper understanding of their regular studies, which may contribute to improved academic achievement when they return to their home campus. Students participating in these experiences tend to gain immersive cross-cultural exposure, incorporating advanced intercultural competencies that foster personal growth and promote global citizenship [3, 22, 23]. However, these numerous positive aspects of international mobility programming also come with difficulties in adjusting to a new academic and cultural environment, causing a common narrative of culture shock experiences [24].

The concept of culture shock was first introduced by Oberg in 1954 to describe the sudden disorientation, anxiety and loss of familiarity experienced by individuals when entering a new cultural environment [25–28]. However, Cupsa [29] describes culture shock as a "transformative process" that can occur both at an individual and societal level as people confront new cultural norms, prompting sojourns to ask the age-old existential question of "Who am I?” The psychological and identity crisis-driven implications of moving from home to the host country can be somewhat of an isolating experience [30, 31], even if the choice to study abroad is an autonomous one.

Schartner and Young [32] state that being an international student is more than just the duration spent in the host country; they reiterate that the experience starts when a student chooses to study abroad. Students might have prepared themselves for this journey by watching movies, learning the host country’s language, or attending pre-departure sessions conducted by their home universities before departing [33, 34]. Oberg’s original work describes the seven stages of culture shock or ‘“adjustment”. as 1. incubation stage, 2. crisis resulting from normal daily activity, 3 understanding the host culture, 4. objective viewing of the host culture. 5. Re-entry, 6. reverse culture shock, and 7. readjustment to the home country [35]. While Oberg is credited with introducing the concept, many researchers have helped to shape the current narrative of between 1–4 stages [36, 37]. The US Bureau of Educational and Cultural Affairs Exchange Program [38] defines the stages as:

- Phase I- The Honeymoon (*individuals tend to be filled with enthusiasm and excitement*, *eager to learn all they can about their new environment*),
- Phase II- The Rejection (*Individuals may feel overwhelmed navigating unfamiliar routines and have an idealized perception that life back in their home country is better and experience a rollercoaster of emotions from anxiety to frustration and even depression and lack of motivation to continue the experience)*, and
- Phase III-The Recovery (*while the journey of adapting to the new environment is never-ending*, *the individual may start to feel as if life is less stressful and start to develop a renewed interest and appreciation for the cultural environment)*

Previous studies have shown that international students frequently experience culture shock when facing challenges such as language barriers, adapting to social norms and navigating unfamiliar academic structures [30, 39], consequently there is a transition phase that students navigate.

### Theoretical framework

Transition Theory has been posited as a framework for understanding individuals’ coping with significant life changes or transitions. These transitions can include going to college, starting a new job, moving to a new city, starting a family as well as non-events-changes that do not occur [40]. Bridges’ Model of Transition [41] makes a lexical case between the meanings of change and transition that can generate pronounced transformative moments in one’s life. Bridges defines change as an external force that happens to people, usually without warning, and defines transition as the internalized psychological shift in processing information at a gradual pace. This suggests variable levels of personal control in relation to change and the consequent individual impact. Piaget’s and Vygotsky’s theories of human development detail that while some changes and transitions are expected, others may be less so. The proposition is that application of the model can provide a framework to help individuals as well as organisations deal with any personal emotional challenges associated with change. In the case of the pandemic, the model fits well since the advent of the pandemic led to enforced changes in the field of student mobility. Therefore, personal control in relation to decision-making was limited, in contrast to the managed change associated with moving abroad to study pre-pandemic. Even though Schlossberg’s transition theory mode [40] is primarily focused on adult transitions [42, 43], it provides a broader overview of transition. In her theory, the transition process includes changes that are anticipated, such as those faced in the context of students deciding to participate in international exchange in a new cultural environment. Thus, it as resonance when exploring the changes international students experienced. Unlike Bridges model, Schlossberg’s transition model states that transition can happen in response to any event, anticipated or unanticipated, as well as in response to a non-event that results in changed relationships, routines, assumptions, and/or roles. Schlossberg’s Theory also emphasizes how perception impacts one’s personal and proactive success during a transition [40].

Transition theory can thus be used to frame the challenge or the navigation process students may face as they adapt to a new education setting. It is not uncommon for students to experience an uncomfortable liminal space of uncertainty in a novel learning environment [44, 45]. Land et al. [8] noted that the transition into higher education after secondary school involves an inevitable process of unlearning, learning and transition, constituting an individual journey in and through higher education. Schlossberg [46] regards this phase as Moving In, Moving Through and Moving Out. Unlearning can be a particularly difficult process as students are expected to encounter ideas that may go against what they have always known to be true [47, 48], which makes self-reflection an integral part of the psychological change/transition process as it allows one to move from experiencing a situation to attempt to understand it [49, 50], with the transition being viewed as subjective [23, 51]. Notably, it explores the psychological experiences individuals undergo when transitioning from one developmental life cycle to another, such as the process of transition that international students encountered when moving from the anticipated in-country experience to online learning during the pandemic.

A criticism of Schlossberg’s theory (which includes her 4S’s model of coping: Situation, Self, Support and Strategies) is that it does not emphasize the social and cultural experiences associated with dealing with change [52]. It also focuses on a Westernised definition of coping that does not reflect a diverse population of learners [53, 54]. Wood et al. [55] noted that transitions into adulthood are more likely to be sensitive to social status, outside forces, and peer relationships and have heightened emotional responses as the desire to belong to groups is prominent. Moreover, cultural differences exist in how people cope with stressful situations, so the coping strategies available in the transition to higher education may not be universally applicable [56, 57]. Social and cultural factors, such as gender, race, class, and other forms of identity, can significantly impact how people experience and navigate transitions [58, 59]. Accordingly, insight into the individual lived experience of international students during the pandemic can inform future global mobility and exchange programs to facilitate transition when faced with ambiguous situations, extending Schlossberg’s theory into a broader social and cultural space.

## Present study

Using Schlossberg’s transition theory, the unanticipated pandemic significantly impacted the anticipated event of studying abroad for international students. Students could not travel freely because of pandemic-related safety restrictions. A wealth of literature examines the international student transition from home to the host country during traditional international education programming [6, 24, 60]. Similarly, there is ample literature on the impact of the pandemic on student experiences overall. However, research on the impact of the pandemic on international students’ study plans and their transition into non-traditional study abroad models are limited.

Disruptions in the learning environment have previously been recognized as opportunities not only to explore system changes within education but also to evaluate those necessitated changes [61–63]. Indeed, during the pandemic, learning was moved from in-person to online in many institutions (dependent on individual countries pandemic-related restrictions), and several studies have emerged that evaluated the impact of the virtual learning environment concerning outcomes associated with domestic students [23, 64–67]. The list of outputs is extensive; however, few accounts specifically reflect the pandemic-driven experiences of study abroad and degree-seeking international students. However, Han et al. [68] explored the experiences of postgraduate international students participating in virtual Education degree programs during the pandemic in the United States, which is aligned with the changes many international students faced at the time (i.e., moving to virtual learning). Han et al. highlighted participants’ preferences for verbal online discussion groups but did not explore their wider student experience. When reflecting on the learning experience, it should be noted that their cohort of Education degree students is a specific group that can be expected to take an interest in a discourse on pedagogical theory and practices. So, it was unknown whether students of other disciplines who were more globally dispersed shared similar experiences in relation to their learning. Therefore, this current study aimed to focus on the psychological and psychosocial experiences of a broader population of undergraduate and postgraduate international degree-seekers and study abroad students whose institutions moved from in-person to online teaching during the pandemic. In this article, the authors use the terms virtual learning and online learning interchangeably since they have delivery approaches that are for the aim of this study sufficiently similar. Data from in-depth interviews offered individual accounts [69] which were analysed using Interpretative Phenomenological Analysis (IPA).

## Methodology

Descriptive semi-structured remote interviews were conducted from 1 February 2021 to 30 June 2021, examining participants’ lived experience of transitioning to online learning during the pandemic. Interpretative Phenomenological Analysis (IPA) is a qualitative analytical approach within psychology based on the underpinnings of phenomenology, hermeneutics, and idiographic philosophy. It focuses on exploring the subjective experiences of individuals, and in this study, the participants’ comments offered a snapshot of the effects of the pandemic on international mobility programs during the first 18 months of this phenomenon. Participants lived experiences and engagement with ’Dasein’ (Heidegger’s 1927 term for ’being in the world’) and the existential meanings they placed on their existence in the new pandemic world were explored. This research was approved by the Research Ethics Committee at Abertay University in Dundee, United Kingdom *EMS3724*.

### Design and participants

In-depth interviews were conducted in the Spring of 2021 as part of a research project conducted for a PhD thesis of the first author. Ten-question prompts *(see Interview Script Prompts abridged version*) enabled participants to recall how they navigated the transition to a different learning environment during the pandemic. Interview questions were specifically devised for this study informed by transition theory and study abroad literature as well as practitioner-orientated conversations and observations made by the first author during the early stages of the pandemic. Ryan and Deci’s (2006) [70] Self-Determination Theory also guided the development of these questions, specifically the aspect of autonomy.

### Interview script prompts abridged version

- What events led to your decision to study abroad in the host country? Can you describe them?
- What did you imagine that Study Abroad would have been like in the host location?
- What experience(s) motivated you to go to college/university in the first place?
- Have you decided to go abroad on a full Virtual Exchange program or a part-time Virtual Exchange? How did you make that decision?
- Can you remember a time when you worked with people from another culture? Please describe how you felt during that time.

The next few questions concern moments that were noticeable or important in both a traditional classroom setting and Virtual Exchange classroom settings

- Are there moments that stand out in your experience of a Virtual Exchange classroom setting?
- What interactions have you had with other participants in the Virtual Exchange classroom?
- What interactions have you had with the facilitator of your Virtual Exchange classroom program?
- What advice would you give to someone who is planning to embark on a Virtual Exchange program?

### Interview prompts included

- Can you describe the feeling for me?
- Why was that important to you?
- What do you mean by that?
- Can you think of a moment that stood out in the process for you?

### Participants

Participants were recruited through three channels: social media (e.g., the Facebook group forum for the School for International Training), written communication (that prompted the research project to several Study Abroad offices in the US and Europe), and snowballing (which asked confirmed participants if they would share the Social Media Call or post within their social circles). The sample was purposive in that participants had to be either degree seekers or study abroad students enrolled at an international higher education university program during the pandemic. A ‘*degree-seeker*’ is defined as someone who has obtained or is in the process of obtaining a formal degree at a host institution different from their country of origin, and ‘*study abroad*’ is defined as a university student studying at a host institution at some point during their formal education while receiving credits at their home institution. Participants included those able to study abroad in a traditional program in Fall 2019 and transitioned online in Spring 2020, those enrolled in hybrid or blended programs that combined in-person programming with online programming and those unable to begin their traditional mobility programs abroad, so the experience was entirely virtual. Using this approach, thirteen undergraduate and postgraduate international students, consisting of 9 females and 4 males from across four continents (*North America*, *South America*, *Europe and Asia)* who self-selected to participate in the study Table 1.

**Table 1:** Profile of participants—international students and demographics.

Gender	Major	Ethnicity	Year in School	Program Type
Female	Humanities	Hispanic	Post-Graduate	Degree-Seeker
Female	Non -Humanities	White	Undergraduate	Study Abroad
Female	Humanities	Hispanic	Post-Graduate	Degree-Seeker
Female	Non-Humanities	White	Undergraduate	Study Abroad
Male	Humanities	White	Post-Graduate	Degree-Seeker
Male	Non-Humanities	White	Undergraduate	Study Abroad
Female	Non-Humanities	White	Undergraduate	Study Abroad
Female	Humanities	Asian	Post-Graduate	Degree-Seeker
Female	Humanities	White	Undergraduate	Degree-Seeker
Male	Humanities	White	Post-Graduate	Degree-Seeker
Male	Non-Humanities	White	Post-Graduate	Degree-Seeker
Female	Non-Humanities	Middle Eastern	Undergraduate	Study Abroad/Degree -Seeker
Female	Humanities	Korean American	Undergraduate	Study Abroad

***Note:***

A code replaced participant names and the specific locations such as country of origin and host country were excluded. *IS-#*, represents International Student and # was the number given to the IS in the order they were interviewed by the first author.

### Procedure and data collection

Semi-structured qualitative interviews served as a means of data collection. *Participant Consent Forms* were provided to participants and once returned, a copy of the *Interview Script-Prompts* was emailed to participants. Each interview was conducted using the Interview Schedule and the prompt questions (*see Interview Script Prompts abridged version in the previous section*). Theories of self-determination, motivation, and transition informed the interview questions, and two pilot interviews helped shape the question’s order and means of delivery. The pilot studies allowed the first author to become familiar with the overall flow of questions. They also provided an avenue to practice the pattern and speed of speech and Microsoft TEAMS was adopted. Using Microsoft TEAMS, thirteen audio/video interviews spanning between 30 and 90 minutes were conducted and recorded online with students who participated in degree seeker/study abroad programs between Fall 2019 and Spring 2021. Data saturation, commonly used in qualitative research, is described as the point at which the researcher feels no new information or themes may emerge [71, 72]. Francis et al. [73] noted that the sample size of participants depended on many factors, and there is no ‘right’ size. However, Smith et al. [74], posit that eight is the suggested number of respondents for saturation using Interpretative Phenomenological Analysis, in recognition that this form of qualitative research can be rigorous and time-consuming. In relation to this study, the first author chose to interview all subjects who volunteered to participate noting that this went beyond what Smith et al. [74] proposed saturation threshold.

Interviews were transcribed, and in line with the Interview Transcript Review process, participants were given copies of the transcripts to allow them validation and alleviate power dynamics, giving participants a choice in what was shared [75].

Participants were given a week to review the transcript, then the first author began the analysis if the participant noted no further comments or concerns. Transcripts were redacted in areas that identified students’ specific geographic locations and excessive filler words were removed. The analysis took several months during which the transcripts were read, listened to and reflected upon several times in an iterative process, which is in line with the IPA method.

The first author had to be mindful of her role in the research process, as she has been a practitioner in the field of international education for more than a decade and was herself an international master’s student at the start of the pandemic before becoming an international PhD candidate. Participants had prior knowledge of her international student status. However, they were only sensitised to her International Education Professional Experience if it specifically arose as a topic during the interview. The practice of reflexivity and compartmentalisation was thus key during the collection and analysis of the data [76]. For that, the first author kept a bulleted journal and received support in confronting assumptions by dialoguing this with the research team thus helping to keep the focus on the participants’ voices.

### Data analysis

Smith, Flowers, & Larkin [74] provide a 1–6 step guide to how to conduct an Interpretative Phenomenological Analysis. This allowed the participant’s lived experiences in their personal and social environments during the pandemic to be explored Table 2 and enabled greater reflexivity on the part of the first author.

**Table 2:** Guide to IPA–a flexible guide to the use of Interpretive Phenomenological Analysis.

Guide to IPA
1. Reading and re-reading
2. Initial noting
3. Developing emergent themes
4. Searching for connections across emergent themes
5. Moving to the next case
6. Looking for patterns across cases

For example, using an excerpt from the transcript of participant IS-13, the first author placed completed transcripts into a table thus allowing the researcher to bracket personal thoughts and emotions when analysing Table 3 The transcribed interview was placed in the centre of the table as a reminder to keep the individual participant at the centre of the analysis. The transcripts were placed into numbered sections, and that section of the transcript was reviewed. This adopted structure enabled comments to be placed in both the *Emerging Themes* section and the *Reflections* section. Steps one to five (*see* Table 2) involved reviewing participants’ transcripts and diligently and repeatedly listening to the audio recordings.

**Table 3:** Transcript excerpt from IS-13 as an example of IPA.

EMERGING THEMES	TRANSCRIPT	REFLECTIONS
**Section 11**	**Participant**	**Section 11**
• She had many options for Summer 2020 ⚬ It was an option, there are many other summer opportunities, but I personally wanted to expand my language skills in *[Host Language]*.	**IS-13:** *It was an option*, *there are many other summer opportunities*, *but I personally wanted to expand my language skills in [Host Language]*. *So that’s why I applied and then I got accepted and took part in the program*.	• Weighing options for Summer 2020 may have allowed the student to feel as if they had some control over her life.

The Findings and Discussion section articulates the outcomes from Step 6, ’Looking for patterns across cases’.

### Findings and discussion

Four primary meaning units and four secondary meaning units were identified. The four primary meaning units were: Functioning in Ambiguity, The Importance of Language and Culture, Reshaping Community and Alternate Experiences in Intercultural Connectivity Table 4.Of the secondary meaning units, the elaboration of *Space* has been the focus of this manuscript due to the prevalence of this meaning unit in the data and its linkage to Schlossberg’s Transition Theory. This research used the categories of primary meaning units as it provided an overview of the meanings derived directly from the participants’ responses. The secondary meaning units in this research are connected to the first primary meaning unit, "Functioning in Ambiguity: Personal and Informational Uncertainty. These secondary meaning units served to organize the broader topic of uncertainty using an existential lens to understand the participants’ lived experiences. To support the organization of the participants’ quotes, Ruonan & Al-Shaibani [77]’s process of using Excerpt 1, 2, and so on was adopted. Notably, the meaning units were interrelated (i.e., can be categorized under more than one primary meaning unit), which is also addressed below.

**Table 4:** Primary and secondary meaning units.

Primary Meaning Units	Secondary Meaning Units
**Functioning in Ambiguity: Personal and Informational Uncertainty**	• **(SPACE) Maintaining Visibility in a Virtual Space** • (TIME) Perception in an unstructured reality • (SUBJECTIVITY) Virtual learning: Perceptions of Benefits and pitfalls • (EMBODIMENT) Embodied expressions of dealing with ambiguity
**The Importance of Language and Culture**	
**Reshaping of Community**	
**Alternative Experiences in Intercultural Interconnectivity**	

### Functioning in ambiguity

The participants expressed experiencing different types of uncertainty, at times simultaneously. These were related to informational (i.e., where information was missing to make an informed decision) or personal uncertainty (i.e., where individuals were aware of the information but unsure if they could perform the task themselves), as distinguished by Van den Bos [78]. For example, participants noted that rules seemed to change daily at the beginning of the pandemic, leaving them with a lack of information and thus unsure about the steps they needed to make in transitioning to a different way of life and virtual learning. An occurrence that Schlossberg calls an unexpected and unanticipated event.

*Excerpt 1 ((IS-2)*

*“*… *my in-person exchange was cancelled* … *I could not redo an exchange later*, *so this had really been my last chance* …*Often your fellow students are still curious about you but making a connection with friendship in mind becomes more difficult behind a screen*.*”*

The above quote also highlights that online connections were perceived as more challenging than in-person connections despite students’ desire to connect. The pandemic created informational and personal uncertainty that disrupted the acculturation and adjustment potential of traditional in-person study abroad experiences [79], as making online connections was perceived as more difficult for some participants. IS-8 was one of the students who began her program in the Fall of 2019. She discussed the difficulties of connecting with other students online, specifically when seeking information, as she felt physical proximity would have aided her understanding.

*Excerpt 2 (IS-8)*

*“*…*in the class like we sit next to each other*, *and we can talk to each other freely*, *for example*, *when there is anything*, *I couldn’t understand*, *I can ask*, *right away*, *the person next to me what did he mean or what do you think about this*? *Also*, *there were a lot more interaction among us but in the online class there’s more interaction between the students toward the teacher but there weren’t many chances for the students to interact*, *to communicate with each other*.*”*

Through the lens of Schlossberg’s theory, it could be perceived that the unanticipated pandemic brought an unexpected dynamic to the teacher-student relationship; however, it also disrupted the student-to-student relationships that might have otherwise occurred in a pre-pandemic traditional classroom. Thus, resulting in another unanticipated change in transitioning to the perceived ambiguity of the virtual classroom.

The secondary meaning unit ‘*Space’* is of particular interest because of its dominance in the data and the physical contact restrictions that were in place in each of the participants’ host countries. The spaces where participants interacted and learned from one another changed into more socially isolated environments. It could be concluded that lockdown and social distancing detracted from the social contact enrichment element that is otherwise an integral part of international mobility programs [22]. However, at the beginning of the pandemic, some participants attempted to navigate opportunities for visibility in their new learning spaces whenever possible.

For example, IS-2 reported how she developed strategies that modified the situation.

*Excerpt 3 (IS-2)*

*“I started facilitating* …*student interaction myself*, *asking people questions*, *and slowly making friends*. *Often*, *I’d talk with people* …*in private chats or we’d hang out more in feedback sessions* … *Showing your face can really help*, *as you become more of “a person” to people*, *therefore easier to interact with*.*”*

IS-7 had a similar experience and decided to be *’seen*’

*Excerpt 4 (IS-9)*

*“I would turn on my camera* …*that would feel nice*. *It would feel like a cozy safe space in my opinion* … *especially when you’re put into breakout rooms and just sit with your teammates and then the lecturer comes in and he has his camera on*, *and we all have our cameras on*. *In my opinion*, *that was nice*.*”*

Initially, international students in this study displayed a degree of flexibility, optimism, and resilience in adjusting to the new virtual learning environment and expressed a desire to exhibit their individuality. The ‘being seen’ element was initiated by some of the international students; however, a number ultimately adopted the methods used by domestic students in their courses, including turning off their cameras and using the chat function.

*Excerpt 5 (IS-11)*

*“*…*I did in the beginning [chat and video]*, *but then I*, *you know*, *I just became tired because no one was doing it*, *and then it’s pretty weird because it’s obviously not the same feeling cause you’re not seeing each other’s eyes and you don’t really know if they’re laughing at you or you know*, *like*, *are they fully listening to you*.*”*

As evidenced in this section, visual (seeing others on camera) and verbal communication was a strategy that the participants perceived as helping to create an inclusive virtual learning environment and aided in the transition process by filling the void of a lack of physical connection. Dryden et al. [80] indicated that language barriers and consequentially cultural differences amplify emotional insecurities, such as feelings of isolation when first faced with a new environment.

### The importance of language and culture

The use of language was vital for participants’ understanding of their host countries and cultures and for connecting the two.

*Excerpt 6 (IS-2)*

*“It was very funny ’cause when they started talking the first week*, *I couldn’t understand them ’cause some of them had like the biggest [Host Country] accents I’ve ever heard*.*” [laughing]*

IS-6 had a similar experience but as a native English speaker.

*Excerpt 7 (IS-6)*

*“I didn’t have to learn [Host Language] cause their English is absolutely phenomenal*. *Like sometimes they’re saying things that I don’t even understand*. *I’m like what’s going on here*. *[Laughing]*

One’s cultural identity can be deeply rooted in language proficiency and an enabler in establishing one’s self-identity [81]. For some people, practice and fluency in the target language while abroad is a source of laughter; for others, it is a source of frustration [82]. The primary meaning unit of ‘Functioning in Ambiguity’ is also present in language usage, as described by IS-3 and her temporary loss of self-confidence in language proficiency.

*Excerpt 8 (IS-3)*

*“I was very scared about not understanding the English I was like; oh my God is my English good enough*? *Am I gonna be able* …*to follow*? *Am I going to be able to write essays*?*”*

The participants in this current study were diverse in their language and cultural understanding; four were native English speakers, and nine were additional English speakers. Although the first two participants found humour in their language discrepancies, it took a little longer for IS-3 as she expressed a keen sense of anxiety concerning being surrounded by a new language and cultural community. Her apprehension of being understood and making connections with others was deeply rooted in how she felt about herself.

*Excerpt 9 (IS-3)*

*“(I was) Scared*, *completely homesick and very lonely*. *I started to make friends*, *but I didn’t feel specifically connected to anyone*. *That only went away when I actually*, *met a friend from [Home Country] and then that changed*. *I think because I needed at that point*, *I needed a connection from someone from home*, *anyone*.

These findings thus expand on Schlossberg’s work in that transition may be complemented by a more nuanced globalized perspective on communication norms when entering a new cultural community. It is essential to understand these cultural differences in order to communicate effectively across cultures [83].

Exposure to the host countries’ language is also an important part of the international student experience when studying abroad [84]. During the pandemic, many participants were isolated from hearing the host language (as the chat-box was predominantly used). The restrictions also reduced informal contact and impromptu interactions with other international students. Other international students are often a primary source of practising host communication skills as conversing with host country locals can feel uncomfortable [85]. The opportunities to engage in activities supporting language development were felt to be more limited; as reiterated in the Functioning in Ambiguity section, the spaces in which they would traditionally interact were altered. It is of note that participants attempted to construct interactive opportunities within their scope of space to help connect with their learning communities.

IS-7 demonstrated the essence of a non-event in her quote below. She had anticipated and planned for an experience to expand her individual growth opportunities and build new connections. However, the pandemic reduced her initial excitement about engaging in the study abroad activity into one of disappointment.

*Excerpt 10 (IS-7)*

*“*… *it was kind of awkward over zoom* … *I was looking forward to* … *getting to meet people and getting to* … *make friends with people that aren’t* …*directly from my social networks*. *So*, *that kind of sucked cause it was very limited* …*being able to talk and meet with each other*.*”*

IS-11 also looked forward to interacting with people outside his existing social network. Although he was able to attend his program abroad, he had more restricted opportunities to experience the language and cultural immersion of his host community.

*Excerpt 11 (IS-11)*

*“I only had the chance to speak with two or three people that were living in my flat* …*at the same time we didn’t talk much because one was really* …*Covid risky* …*they didn’t take much risk about going outside of the room and then the second one*, *we did talk a bit*, *but we didn’t have much in common*.*”*

The challenge of international students entering a new language environment is a documented phenomenon in studying abroad [86]. However, Covid restrictions and the move to virtual spaces meant that participants lacked the opportunity to practice social and adaptive skills in their new language and cultural environments.

### Reshaping of community

International students, like first-year university students, are trying to find their place in an unfamiliar communal learning space [87]. As emerging adults in this context, it can be expected that they were trying to find a sense of self and how they might fit into the social world [e.g., 55]. Unlike first-year university students, who often start in cohorts, this study’s participants were sometimes the only new enrolment on a study stage as they began their exchange experiences at different points before, during and beyond the initial pandemic announcement.

Participants who began their international programs in the Fall of 2019 as degree-seeking or study abroad reported that they had built face-to-face relationships before delivery moved online. These pre-pandemic relationships provided a sense of community, enabling them to work more effectively in the virtual classroom. This sense of community and the effective use of online learning tools were interdependent in the students’ experiences. The value of community contacts emerged in the students’ narratives as these supports enabled bouts of positive transition.

*Excerpt 12 (IS-5)*

*“… We were a few people; we were like 10 to 11 people … We all turned on our cameras and it was nice*. *Yeah, because we knew each other so we felt comfortable”*

*Excerpt 13 (IS-7)*

*“*…*as the course continued*, *probably like a month or so* …*we had like a group chat and we were like talking more*, *you know*, *like making jokes as like anyone does in [a normal] classes*, *just trying to get along throughout the course* …*”*

Students in the first group, who had begun studying abroad before the pandemic, stated that after their first semester in their host countries, they felt more comfortable in their new communities, which led them to feel less apprehensive going into Spring 2020. IS-6 used the word ‘*chaotic*’ on several occasions when describing his experiences abroad but tended to use it positively when discussing his adjustment period.

*Excerpt 14 (IS-6)*

*“I felt like I was [a part of Host culture]* … *You should see how chaotic it is (riding a bike in host country’s “massive cycling culture”)* …*honestly*, *it’s absolutely nuts* … *because of the chaos it was kind of fun to be in that atmosphere quiet*, *like*, *chaotic atmospheres*.*” [Laughing]*

IS-12 also stated that her level of comfort had increased at the start of her second semester abroad but expressed a sense of disappointment at the onset of the pandemic.

*Excerpt 15 (IS-12)*

*“The Spring semester*, *crushed dreams*, *[Laughing]… [start of Spring 2020] we knew everything by that point*. *We knew how the school system worked*. *We knew a lot of the lecturers and we knew our way around the city*. *We were settled and it was so nice*. *It felt like another level of freedom* …*you’re still in a new place*, *but it doesn’t seem strange anymore*. *It feels like you are part of the community* …*”*

IS-1 had established in-person connections with her classmates in the Fall of 2019, therefore she was able to draw on these prior connections in the virtual space, albeit with some adjustments.

*Excerpt 16 (IS-1)*

*“*… *I know my classmates really well; we bonded really well*. *We were actually friends* …*I was able to go online and you’re going to breakout rooms*, *we would talk just* …*you know with each other*, *and we would run jokes and we can open a document and Google Doc shared and do our work*, *you know and present together*. *We found a way*, *you know*, *to present our work* …*do it together* …*”*

There were three Summer 2020 participants were from military communities and were the only ones who participated in a language-based program that was entirely virtual prior to the commencement of their core program. One of the participants described the difficulty she faced with learning a new language online and making connections with her peers on the program.

*Excerpt 17 (IS-4)*

*“Honestly*, *online it was a little awkward at first because like we just went on to the zoom and then like the teacher just ended it and then we like*, *nobody had time to exchange contact information over the first like week and a half*. *We kind of just logged off and left*, *until somebody finally like*, *made a group chat*. *But then we became friends cause we talked a lot*, *but it was rough*.*”*

While these three participants came from different military branches, they shared a common bond which may have been an impetus to support to one another. They worked together to complete their international program and, in turn, learned to build a new community. For IS-12 being needed by and supporting others enabled her to develop new communities.

*Excerpt 18 (IS-12)*

*“I started volunteering at a cafe for women to help them with mental health* …*we talk about everything*. *I started volunteering there and it was mostly because I started to notice how a lot of people are actually struggling because of Covid*….*I met a lot of different people there and it felt good* …*I don’t think anyone sees as many people as I see in a week [laughing] because I also work at a gym for elderly women* … *it’s a community*, *everyone knows each other*.*”*

Operating in a safe communal environment can build internal self-determination and resilience. This has been noted to be present in internationally mobile students [45, 88]. It is in this communal environment that shared knowledge and intercultural structures can be practised and built, especially in the case of international students as they navigated new learning environments in *‘normal’* circumstances prior to the pandemic, as discussed by Wu et al. [89] and Taylor & Ali, [6]. Due to the pandemic, traditional methods of community building were restricted. However, most participants reported that they were able to reshape their understanding of what community meant to them. Various dynamics were involved in reshaping communities between participants of this study.

Participants who had previously experienced traditional education abroad, such as travelling from home to the host country and physically interacting with peers, appeared to more easily transition to the online community. This insight could have relevance to higher education communities as hybrid/blended programs emerge from pandemic course structures. Pandemic-associated ambiguity, cultural misunderstandings, and reestablishing a community online may not have been the optimal scenario for a successful international education experience. However, students found new ways of adapting to change during a period of personal and informational uncertainty.

### Alternate experiences in intercultural interconnectivity

Feelings of frustration concerning not being able to have the perceived full experience of spending time outside one’s home country were expressed repeatedly. Particularly strong was the notion of a loss of cultural experiences, showing that intercultural opportunities are an underlying motivation for studying abroad, as evidenced in the two examples below:

*Excerpt 19 (IS-3)*

*“It’s not just going to the [classes], it’s also being able to explore the city you’re in. That was one of the most beautiful things for me. Being able to explore restaurants in the street and the people and nature. If you cannot do that, or if you’re afraid to do that either for your own safety or for the laws that the country you’re in has …it doesn’t give you the whole experience.”*

*Excerpt 20 (IS-13)*

*“If I was actually in [Host Country]*? *I think I would actually be able to experience it*, *taste the food or take part in cultural activities and like actually talk to the people that live there*.*”*

IS-13’s reference to her desire to interact with the host community on more than just a cursory level returns the reader to the premise that cultural understanding is a crucial component of the

communicative experiences that one can associate with, being a part of the social world.

During the pandemic, many of us were forced to confront uncertainty. This process, while challenging, also presented an opportunity for personal and professional growth. As Schlossberg [10] suggests, by reevaluating non-events, we can reimagine our future. This was particularly evident in the way individuals coped with their ability to be understood and cultivated a sense of community. These efforts not only required personal resilience but also provided a platform for reflecting on future career options.

One such example is IS-10, who expressed how he could envisage his future professional life based on his online study experience.

*Excerpt 21 (IS-10)*

*“*…. *I think it’s how I see myself working in the future*, *either purely remotely or mostly remotely*, *just because of the practice [lockdown and virtual classes]*. *I now have this idea of what working from anywhere in the world would be like and I think that would align very well with working remotely because I don’t want to go to work every day*.*” [Laughing]*

The facilitator ‐ student dynamic also played a role in building a new secure community where open dialogue was encouraged.

*Excerpt 22 (IS-3)*

*“I really appreciated [facilitator] asked me directly…*. *‘I think you’re the only international student that is doing this class, how are things for you? How are things at home? Can you leave, and for me that made all the difference’.”*

*Excerpt 23 (IS-10)*

*I’m really grateful that teachers* …*for the lectures themselves*, *they did try to do their best* …*I feel like everything has been really productive* … *the structure was followed and I find it really clear* …, *like to follow classes and work on the assessments and* …*still get in touch*, *despite that*, *I’m not actually*, *physically in classes*.

Adjustments had to be made in the learning environment and a number of participants noted the importance of dialoguing with their facilitators and noted their assistance was essential to navigate an ambiguous time.

## Conclusion and limitations

Participants in this study had expected to travel to their destination of choice, experience a new culture and meet personal and professional challenges when they first self-selected their study abroad/degree seeker destinations. Yet the pandemic brought disruption to everyday life around the world. The international students who participated in this study were active in finding their ‘*cultural fit*,*’* meaning navigating new ambiguous spaces in language and culture and reshaping what community meant to them, despite the pandemic. This finding shows levels of resilience in international students which are discussed below. Students also experienced a change in teacher-student and student-student relationships and noted the importance of the spoken language in international exchanges.

Schlossberg’s Anticipated, Unanticipated, and Non-event transition types provided a lens through which to view the lived experience of international students. In the context of the pandemic, international students faced numerous changes to their anticipated time abroad. This research documented their transformation from uncertainty through resiliency in the face of their pandemic-driven non-event experiences. Participants reported developing different strategies to engage in their programs as well. The expectation of a need to adapt to change in a new physical environment (the host country) prior to the pandemic may also have helped these international students cope with changes in their virtual environment. That is, they had already anticipated a need to do things differently and build new relationships in an unknown context. They had to learn to function in an online society because of the pandemic guidelines, but their desire to build human connections remained and transferred to the virtual world [90]. Additionally, several participants seemed to rely more heavily on facilitators in the learning spaces, i.e., International Education Professionals, to help them with the changes. Facilitators were perceived as essential to classroom community and connectivity in virtual courses. Notably, these interactions, such as expressing empathy by inquiring about the participants’ health and family concerns during the pandemic, allowed rapport building with others, which Kaufmann and Vallade [91] highlighted as helping to alleviate the perceptions of loneliness.

This study revealed language as a critical component of international student development and should be remembered in the virtual classroom. Whilst some participants took part in an international language exchange program, the language immersion was an experience most participants talked about. Language competency can strengthen one’s self-identity, increase intercultural knowledge, and enhance connections to a chosen study-abroad destination. The social isolation of the pandemic did not enable the expected host community interactions that are usually associated with international student mobility programs. Furthermore, Han et al. [68] stated that ‘linguistical and conceptual misunderstanding’ could potentially hinder or decrease an international student’s comfort and inquisitiveness in virtual space. A sentiment corroborated by the participants in this current study. The lack of a sense of normalcy in the virtual setting also required individuals to redefine community, as well as a adjust to their sense of loss of opportunities. It is important to note that language can be a valuable tool in building community and connecting with others in society, culturally and personally [81]. One limitation of concern in this study is that all participants were either westernized or studied in Western cultures. Efforts were made to recruit a more diverse population of international students who could have provided a broader global view of the pandemic’s impact; however, since participants had self-selected to participate in the study, it was not possible within the given timeframe. Additionally, this study draws on self-reported occurrences influenced by memory recall but illustrates how the pandemic challenged these individuals in study abroad programs emotionally and otherwise. Importantly, it was noticeable that the ‘being prepared’ for changes supported international students in acting proactively in their learning and social networking. A finding worthy of further exploration to better understand approaches to support students transitioning into and throughout Higher Education. Whilst the importance of language and community on individual’s lived experience of international exchanges during the pandemic were this study’s most prominent observations, it is of relevance to note that all three authors originate from different countries and have diverse cultural knowledge. Two authors are native English speakers, one from the US and the other uses a British dialect; the third is an additional English language speaker. All met in the same geographic area, one which has its own regional dialect; therefore, the diversity of the authors’ language experiences brings richness to the interpretative aspects of the participants’ experiences but might also have increased their own attributions with respect to this meaning unit. Nevertheless, as the delivery of online courses has gained momentum in academia, it is vital that the usage of language and cultural encounters is not lost in the transition. Therefore, it could be of significance for institutions to reflect on the exposure to local language and cultural references within discipline-based synchronous and asynchronous pedagogic interactions. However, it may also be of merit to consider the immersive and community-oriented experiences that arise from the curriculum during the term time as well as ahead of it. The anticipation of the upcoming study term can empower both prospective and current students to assemble psychological resources (motivation, resilience) that may enable a smoother transition. By recognizing and studying these experiences, researchers and educators can better support and assist international students during times of uncertainty and change to their educational environments.
